# The complete chloroplast genome sequence of *Podocarpus macrophyllus* (Podocarpaceae) and phylogenetic analysis

**DOI:** 10.1080/23802359.2022.2094294

**Published:** 2022-07-04

**Authors:** Zhi Zhu, Xiaoping Li

**Affiliations:** a Collaborative Innovation Center of Southern Modern Forestry, Nanjing Forestry University, Nanjing, China; b College of Forestry, Nanjing Forestry University, Nanjing, China; c Jiangsu Key Laboratory for Poplar Germplasm Innovation and Variety Improvement, Nanjing Forestry University, Nanjing, China

**Keywords:** *Podocarpus macrophyllus*, Podocarpaceae, complete chloroplast genome, phylogenetic analysis

## Abstract

The complete chloroplast genome sequence of *Podocarpus macrophyllus* was determined in this study. According to the results, the genome is 134,239 bp in length. The GC content of the whole chloroplast genome is 37.0%. The complete chloroplast genome of *P. macrophyllus* encodes a total of 120 genes, including 34 tRNA genes, 4 rRNA genes and 82 protein-coding genes. Like other conifers chloroplast genomes, *P. macrophyllus* has no inverted repeat sequences. To reveal the phylogenetic relationship of *P. macrophyllus*, we constructed phylogenetic trees using other species of Podocarpaceae, and the phylogenetic analysis showed that *P. macrophyllus* is evolutionarily closest to *Podocarpus longifoliolatus*.

*Podocarpus macrophyllus* (Thunb.) D. Don 1818, is an evergreen coniferous tree species of the Podocarpaceae family, widely distributed in South China and Japan. *P. macrophyllus* is versatile, widely used for landscape, medicine, bonsai, etc. It adapts well to shearing so that it can be shaped to your needs. The wood can be used to make furniture because of its high toughness and hardness and its tolerance for low light makes it grow well indoors as a potted plant. Thanks to its low maintenance, adaptability and ornamental, this hardy evergreen plant is an ideal garden landscape tree species. In addition, the roots, bark and leaves of *P. macrophyllus* are also commonly used as traditional Chinese medicine (Qiao et al. 2014; Qi et al. 2018). For further research of *P. macrophyllus*, we sequenced its complete chloroplast(cp) genome sequence.

The fresh leaves of *P. macrophyllus* were collected from the Nanjing City, Jiangsu Province, China (32.05000°N, 118.78333°E) for genomic DNA extraction. The voucher specimens were deposited in the herbarium of Nanjing Forestry University (https://www.njfu.edu.cn/, voucher number: NJLHS2020_008; Xiaoping Li, xpli@njfu.edu.cn). The genomic DNA was extracted by a modified CTAB method (Allen et al. 2006). The whole genome was sequenced on the Illumina HiSeq platform (Illumina, San Diego, CA) and a total of 2.6 G raw reads were obtained. After being filtered by PRINSEQ lite v0.20.4 (Schmieder and Edwards 2011), we finally got 2.55 G clean reads. Then, the complete cp genome of *P. macrophyllus* was assembled by NOVOPlasty (Dierckxsens et al. 2017). The assembled sequence was preliminarily annotated by Plastid Genome Annotator (PGA) (Qu et al. 2019) and the preliminary annotation result was corrected manually. The complete cp genome sequence has been submitted to GenBank (accession number OM302532).

The complete cp genome sequence of *P. macrophyllus* is 134,239 bp in length. Like other conifers cp genomes, *P. macrophyllus* cp genome doesn’t have a typical quadripartite structure due to the loss of an inverted repeat region (Raubeson and Jansen 1992). A total of 120 genes are encoded in the complete cp genome of *P. macrophyllus*, including 34 tRNA genes, 4 rRNA genes and 82 protein-coding genes. The total GC content of cp genome is 37.0% and this value in the protein coding region, tRNA gene and rRNA gene is 38.1%, 53.2% and 54.0%, respectively. Among 14 genes in *P. macrophyllus* cp genome, all contain only one intron excluding ycf3 containing two.

In order to reveal the phylogenetic relationship of *P. macrophyllus* within Podocarpaceae, we chose other 14 species of the Podocarpaceae family to phylogenetic analysis, with *Ginkgo biloba* L. 1771 as an outgroup. The complete cp genome sequences of the 14 Podocarpaceae species, *G. biloba* and *P. macrophyllus* (a total of 16 chloroplast genome sequences, all downloaded from NCBI except *P. macrophyllus*) were aligned using MAFFT v7.453 (Katoh and Standley 2013) and a maximum-likelihood (ML) phylogenetic tree based on 16 complete cp genome sequences was constructed by MEGA v11.0.11 (Hall 2013) with 1000 bootstrap.

The phylogenetic relationships among the Podocarpaceae species were supported by high bootstrap values (Figure 1). The phylogenetic analysis showed that *P. macrophyllus* is evolutionarily closest to *Podocarpus longifoliolatus* Pilg 1903.

**Figure 1.**
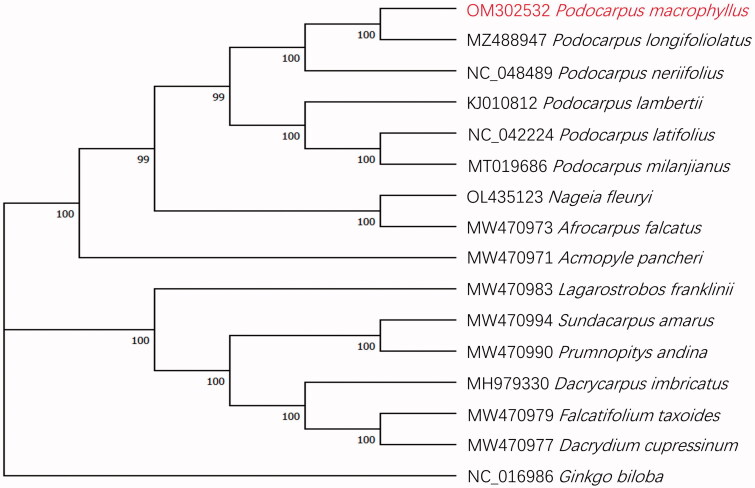
A maximum-likelihood phylogenetic tree based on 15 complete chloroplast sequences of Podocarpaceae species using *Ginkgo biloba* as an outgroup with 1000 bootstraps. Numbers below the branches indicate the bootstrap values.

## Data Availability

The complete chloroplast genome sequence data of *Podocarpus macrophyllus* supporting the findings of this study are openly available in Genebank of NCBI at http://www.ncbi.nlm.nih.gov/ under the accession number OM302532. The associated BioProject, SRA, and BioSample numbers are PRJNA822864, SRR18591619, and SAMN27280562 respectively.

## Regulation statement

This study strictly follows the wild plant protection regulations of China, which were firstly promulgated by Decree No. 204 of the state council of China on Sept. 30, 1996 and amended on Oct.7, 2017 under Decision of The State Council on Revising Some Administrative Regulations.
